# The COMER web server for protein analysis by homology

**DOI:** 10.1093/bioinformatics/btac807

**Published:** 2022-12-15

**Authors:** Justas Dapkūnas, Mindaugas Margelevičius

**Affiliations:** Institute of Biotechnology, Life Sciences Center, Vilnius University, Vilnius 10257, Lithuania; Institute of Biotechnology, Life Sciences Center, Vilnius University, Vilnius 10257, Lithuania

## Abstract

**Summary:**

Sequence homology is a basic concept in protein evolution, structure and function studies. However, there are not many different tools and services for homology searches being sensitive, accurate and fast at the same time. We present a new web server for protein analysis based on COMER2, a sequence alignment and homology search method that exhibits these characteristics. COMER2 has been upgraded since its last publication to improve its alignment quality and ease of use. We demonstrate how the user can benefit from using it by providing examples of extensive annotation of proteins of unknown function. Among the distinctive features of the web server is the user’s ability to submit multiple queries with one click of a button. This and other features allow for transparently running homology searches—in a command-line, programmatic or graphical environment—across multiple databases with multiple queries. They also promote extensive simultaneous protein analysis at the sequence, structure and function levels.

**Availability and implementation:**

The COMER web server is available at https://bioinformatics.lt/comer.

**Supplementary information:**

Supplementary data are available at *Bioinformatics* online.

## 1 Introduction

Establishing homology among proteins is essential to studies of protein evolution and phylogenetics (Buchfink *et al.*, 2021; Dereeper *et al.*, 2008), protein annotation (Mirdita *et al.*, 2017; UniProt Consortium, 2021), classification (Chandonia *et al.*, 2019; Schaeffer *et al.*, 2017; Sillitoe *et al.*, 2021), structure (Baek *et al.*, 2021; Jumper *et al.*, 2021; Kryshtafovych *et al.*, 2019) and function prediction (Zhou *et al.*, 2019). In all these fields, accurate alignments between homologous protein sequences and their abundance are key to accurate and confident inference. Therefore, sensitive homology search tools capable of producing high-quality alignments are of great importance.

To this end, we present a new web server for multipurpose protein analysis based on our recent development of sensitive, accurate and fast homology searches. The homology search method COMER v2 (Margelevičius, 2020), at the core of the COMER web server (hence the name), provides high sensitivity through a comparison of sequence family models (profiles). The sequence alignments that COMER produces exhibit high accuracy, implying that a protein 3D structural model generated using a statistically significant COMER alignment (Margelevičius, 2019) is significantly similar to the native structure. Along with producing high-quality alignments, COMER2 harnesses the power of graphics processing units (GPUs) to accelerate profile–profile comparison and homology searches. Consequently, large protein databases can be searched in seconds.

## 2 Materials and methods

To improve the services the COMER web server provides, we have introduced four enhancements (v2.3) to COMER2 since its last publication (Margelevičius, 2020): (i) masked profile positions (e.g. corresponding to low compositional complexity regions) have been developed so that they do not contribute to profile–profile scoring by default. Previously, masked positions were assigned the amino acid background probability distribution, which produced small non-zero scores for some position pairs. The accumulation of such positive scores led to incorrect alignment stretches. (ii) The automatic selection of GPUs for execution has been modified to skip busy GPUs or GPUs with insufficient free memory. (iii) An algorithm for simultaneously searching multiple databases and (iv) support for the machine-readable JSON output format have been implemented.

The qualities of the COMER method and additional developments provide the COMER web server with distinctive features. The COMER2 software architecture permits simultaneously running multiple instances of homology searches on the same GPU independently without compromising speed (Margelevičius, 2020). Consequently, the web server can efficiently exploit computational resources and distribute workload across multiple dedicated GPUs. Under the current setting, up to four independent COMER2 searches per GPU with 16 GB of HBM2 memory can be conducted at the same time. Furthermore, utilizing the advanced features of COMER2 software, the web server allows the user to submit many sequences, multiple sequence alignments (MSAs) and profile queries in different formats at once. Organizing and processing user queries in bulk remove the limitation of focusing on one protein of interest at a time and thus save the user time and effort.

**Fig. 1.**
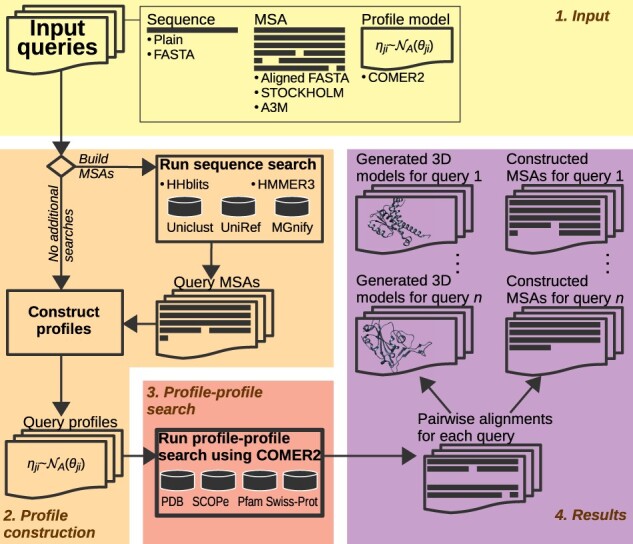
Flowchart for the COMER web server. (1) The user can provide sequences, MSAs and COMER2 profiles in different formats in the same input field. The server automatically determines the format. (2) The server can be instructed to build informative MSAs for user queries (profiles excluded). In this case, the server performs additional sequence searches using HHblits (Remmert *et al.*, 2011; Steinegger *et al.*, 2019a) against the Uniclust (Mirdita *et al.*, 2017) or BFD (Steinegger *et al.*, 2019b) database (not shown) and/or HMMER3 (Eddy, 2011) against the UniRef (Suzek *et al.*, 2015) or MGnify (Mitchell *et al.*, 2020) sequence database. COMER2 profiles are constructed for each sequence and MSA corresponding to a user query. (3) Searching at various levels of protein knowledge is provided by a profile–profile search across different COMER2 databases (several are shown; see text for details). (4) Based on the produced alignments, the user can construct profile–profile-guided MSAs and generate structural models by homology in bulk

The web server permits simultaneous searching across multiple profile databases with user queries. An optional sequence database search with the sequence and MSA queries to increase informativeness can precede a profile–profile search (Fig. 1). The target profile databases are available for analysis for a wide range of levels of protein knowledge (Fig. 1): (i) PDB (Burley *et al.*, 2021) proteins with known structure; (ii) Pfam (Mistry *et al.*, 2021), COG (Galperin *et al.*, 2021; Tatusov *et al.*, 2003) and NCBI’s CDD (Lu *et al.*, 2020) protein families; (iii) SCOPe (Chandonia *et al.*, 2019) and ECOD (Schaeffer *et al.*, 2017) classified proteins; and (iv) UniProtKB/Swiss-Prot (UniProt Consortium, 2021) annotated proteins. The server also simplifies the analysis of results at the residue level by providing the option to construct an MSA based on COMER2 pairwise alignments, which can be selected individually or as a group with estimated statistical significance within a specified interval. For structural analysis, the server can be instructed to generate 3D structural models for query proteins using selected COMER2 pairwise alignments. There are two possibilities: a single structural model, using all selections as restraints, or multiple models, one for each selected alignment. Importantly, structural modeling is also supported based on hits to UniProtKB/Swiss-Prot representatives for which AlphaFold2 models are available (Varadi *et al.*, 2022). Further details can be found in Supplementary Section S1.

## 3 Results and discussion

The COMER web server supplements the available services that support protein analysis by conducting fast profile–profile homology searches (Zimmermann *et al.*, 2018). Compared to the MPI Bioinformatics Toolkit (Gabler *et al.*, 2020), the COMER web server offers the following new features: (i) the COMER2 profile–profile search tool, which has not been available on the web until recently; (ii) profile construction using multiple sources; (iii) simultaneous multiple homology searches and (iv) protein 3D structure predictions; and (v) a RESTful application program interface (API) to provide command-line and programmatic access to the web services. In Supplementary Section S3.1, we also provide a comparison with existing services, including HHpred (Hildebrand *et al.*, 2009) from the MPI Bioinformatics Toolkit, regarding sensitivity, precision, alignment quality and execution time. Supplementary Section S3.2 provides the execution times of the COMER web server for various settings.

The web server offers services for protein analysis at the sequence, structure and function levels (see Supplementary Section S2 for a detailed description of these services). We present an example study of protein annotation supported by the COMER web server. Using COMER2, we searched all 4730 families of Pfam 34.0 domains of unknown function (DUFs) in the UniProtKB/Swiss-Prot90 (2021_03) database and analyzed the number of unique gene ontology (GO) Cellular Component, Molecular Function and Biological Process terms associated with each DUF family through identified significant hits. COMER2 produced 74 506 significant alignments in total. In comparison, HMMER3 (Eddy, 2011), the basic tool for constructing the Pfam database, produced 13 579 significant alignments. The analysis presented in Supplementary Section S4 shows that significant hits that COMER2 identifies generally represent true relationships, and COMER2’s sensitive profile–profile comparison is complementary to and may be useful in protein functional annotation. Two examples given in Supplementary Section S5 demonstrate this potential.

We hope that researchers will find the COMER web server a useful resource with a user-friendly interface, high-quality tools, up-to-date target profile databases and services for protein analysis.

## Supplementary Material

btac807_Supplementary_Data

## Data Availability

The data underlying this article are provided in Supplementary Section S6.
