# Supplementary material for: The COMER web server for protein analysis by homology
Source: Bioinformatics. 2022 Dec 15;39(1):btac807. doi: 10.1093/bioinformatics/btac807 (PMC9825750; doi:10.1093/bioinformatics/btac807)
Supplement: btac807_Supplementary_Data [file btac807_supplementary_data.pdf]

# The COMER web server for protein analysis by homology

## Supplementary Materials

Justas Dapkūnas, Mindaugas Margelevičius*

*Institute of Biotechnology, Life Sciences Center, Vilnius University, Vilnius, Lithuania*

## Contents

**S1 Supplementary materials and methods** 2
S1.1 Workflow of the COMER web server . . . 2
S1.2 COMER2 profile databases . . . 3

**S2 Web server features** 3
S2.1 Input to the COMER web server . . . 3
S2.2 Server output environment . . . 4
S2.2.1 Informative alignments and statistics . . . 4
S2.2.2 Services at the sequence, structure, and function levels . . . 6
S2.2.3 Switching between jobs . . . 6
S2.2.4 Data availability . . . 6
S2.3 RESTful API . . . 6

**S3 Benchmarking the COMER web server** 7
S3.1 Comparison with HHpred and HMMER web services . . . 7
S3.2 Execution duration . . . 9

**S4 Rapid extensive annotation of proteins of unknown function** 12

**S5 Case studies** 14
S5.1 PF11821 . . . 14
S5.2 PF09196 . . . 14

* **Correspondence**: mindaugas.margelevicius@bti.vu.lt

**S6 Software and data availability** 17

**References** 18

# S1 Supplementary materials and methods

## S1.1 Workflow of the COMER web server

The flowchart for the COMER web server is shown in Figure 1 of the main text. The ultimate goal for the server is to rapidly produce accurate pairwise sequence alignments for protein analysis for multiple queries, which the user can submit in various formats. The user can provide sequences in plain text and FASTA format, MSAs (multiple sequence alignments) in aligned FASTA, STOCKHOLM, and A3M formats, and COMER2 profiles—all in the same input field. The server automatically determines the format of the input data.

The COMER web server can be instructed to build informative, diverse MSAs for user queries (profiles excluded) to achieve sensitive, specific results. In that case, the server performs additional sequence searches with user queries using HHblits (Remmert *et al.*, 2012), HMMER3 (Eddy, 2011), or *both*, and builds MSAs from statistically significant hits. The Uniclust (UniRef) (Mirdita *et al.*, 2017) and BFD (Steinegger *et al.*, 2019) databases filtered to 30% sequence identity are available for searches using HHblits, while the UniRef (Suzek *et al.*, 2015) filtered to 50% sequence identity and the MGnify metagenomic (Mitchell *et al.*, 2020) sequence database for HMMER3. When using both of these tools, an MSA for each query results from combining sequence alignments they produce independently, which is useful for query sequences with low homology.

Each sequence and MSA corresponding to a user query is subjected to profile construction. Profiles form the basis for profile-profile searches using COMER2. COMER2 profiles include SS predictions (Jones, 1999) made for query proteins. Searching at various levels of protein knowledge is provided by a profile-profile search across COMER2 databases corresponding to the PDB, SCOPe (Chandonia *et al.*, 2019), and ECOD (Schaeffer *et al.*, 2017), all filtered to 70% sequence identity, Pfam (Mistry *et al.*, 2021), COG (Galperin *et al.*, 2021; Tatusov *et al.*, 2003) and NCBI's CDD (Lu *et al.*, 2020), and UniProtKB/Swiss-Prot (UniProt Consortium, 2021) filtered to 90% sequence identity. All these databases contain SS predictions to increase sensitivity.

The format in which the results of a profile-profile search appear includes for each user query a list of pairwise alignments between the query and a database sequence, both representing constructed profiles. The alignments produced are amenable to further analysis. Their different selection combinations for a query allow the user to construct different MSAs and generate 3D structural models by homology to detected proteins (Webb and Sali, 2016) in bulk when the protein sequences have a known structure. These analyses are available as separate job submissions to the COMER web server (not shown in Figure 1 for clarity).

The SLURM workload manager (Yoo *et al.*, 2003) schedules and dispatches users' jobs for execution. Jobs run on the CentOS 7 Linux operating system deployed on a dual-socket GPU server with 128GB DDR4 RAM and Intel Xeon Gold 5115 CPUs (20 cores, 40 threads) clocked at 2.4GHz, equipped with three NVIDIA Tesla V100 GPU accelerators with 16GB HBM2 memory. Presently, two GPUs are assigned to the COMER web server's processes. This configuration supports a maximum of eight COMER2 searches running in parallel on GPUs, each processing multiple user queries simultaneously. (A homology search is configured to use 4GB of GPU

memory and corresponds to processing all the queries of one user.)

Access to the COMER web server is provided via a secure HTTPS connection. The frontend of the COMER web server is deployed on a Linux virtual machine hosted on a separate computer. It establishes a secure connection to the backend GPU server (where calculations take place) before sending input data and receiving computed results. For maintainability and supporting different web browsers, the frontend is written in Python using the Django web framework and in JavaScript with the Bootstrap 5 framework. The source code of the frontend, backend, and component tools is publicly available (Software and data availability).

### S1.2 COMER2 profile databases

The COMER web server permits simultaneous searching across multiple profile databases. The following target profile databases are available for searching using COMER2: (i) PDB (Burley *et al.*, 2020) proteins with known structure; (ii) Pfam (Mistry *et al.*, 2021), COG (Galperin *et al.*, 2021), and NCBI's CDD (Lu *et al.*, 2020) protein families; (iii) SCOPe (Chandonia *et al.*, 2019) and ECOD (Schaeffer *et al.*, 2017) classified proteins; and (iv) UniProtKB/Swiss-Prot (UniProt Consortium, 2021) annotated proteins.

The UniProtKB/Swiss-Prot profile database is constructed as follows. The corresponding sequence database is filtered to include only sequences of at least two BLAST (Altschul *et al.*, 1997) words in length ($>5$). Then, it is clustered at 90% sequence identity using the BLAST-clust program from the NCBI BLAST software suite with soft masking of low-complexity regions and a length coverage threshold of 0.9 applied to either of the two sequences being compared. COMER2 profiles are constructed from multiple sequence alignments (MSAs) built for the resulting sequences. The MSAs are obtained by running HHblits (Remmert *et al.*, 2012) for each sequence for three iterations against the Uniclust database (Mirdita *et al.*, 2017) filtered to 30% sequence identity and including statistically significant hits ($E$-value $\leq 0.01$). Secondary structure (SS) predictions are calculated from the MSAs using PSIPRED (Jones, 1999) and added to the COMER2 profiles. The COMER2 profile database then becomes the collection of the COMER2 profiles.

The Pfam profile database contains the COMER2 profiles constructed for each Pfam sequence family seed alignment. The other profile databases are the COMER2 profiles constructed for the MSAs from the respective HHpred databases (Zimmermann *et al.*, 2018). All the profiles in the databases include SS predicted by PSIPRED.

## S2 Web server features

### S2.1 Input to the COMER web server

The COMER web server's main page is used to enter one or more sequence, family, and/or profile queries and to specify options for controlling the homology search process. Multiple queries can be entered into the designated edit field or uploaded as an input text file. The separator line "//" indicates the end of an individual query, which can represent a sequence, in plain or in FASTA format; an MSA in aligned FASTA, STOCKHOLM, or A3M format; or a COMER2 profile. The type and format of a query are automatically determined. Currently, the limit for the maximum number of queries for a simultaneous COMER2 search is 100.

The main page provides options for selecting target profile databases. Options also allow the

user to specify how profiles are constructed for queries—that is, whether the queries (e.g., query MSAs) are directly used for profile construction or intended for searches using HHblits and/or HMMER3, followed by profile construction. More rarely used options provide controls that affect the quantitative and qualitative aspects of resulting profile-profile alignments, whose descriptions are given on the help page. All options have default values so that the user can focus on the input data of interest. A sample filled-out job submission form is available on the main page.

Before submitting a job for execution, the user can provide a job name and an email address for notification upon job completion. Once the job has been submitted, a link to the results page will appear. This link can be bookmarked for later use.

## S2.2 Server output environment

***S2.2.1 Informative alignments and statistics*** The main results page of the COMER web server lists the links to the respective results pages for each user query in the same order as the queries appear in the input. An individual results page contains the summary and alignment sections. The summary section graphically displays the query regions aligned with identified target (database) proteins (Figure S1), which are listed along with alignment scores and statistical significance estimates ($E$-values) (Margelevičius, 2019) below the graphical representation. The alignment section shows the pairwise profile-profile alignments between the query and the identified proteins along with the predicted SSs, which help visually assess how well structural features align. (Profile-profile alignments are shown as sequence alignments, with sequences representing constructed profiles.) Alignment statistics that accompany each alignment provide additional criteria for assessing the closeness of the aligned pair (e.g., sequence identity percentage) or compositional similarity (relatively low values of lambda (Margelevičius, 2019)).

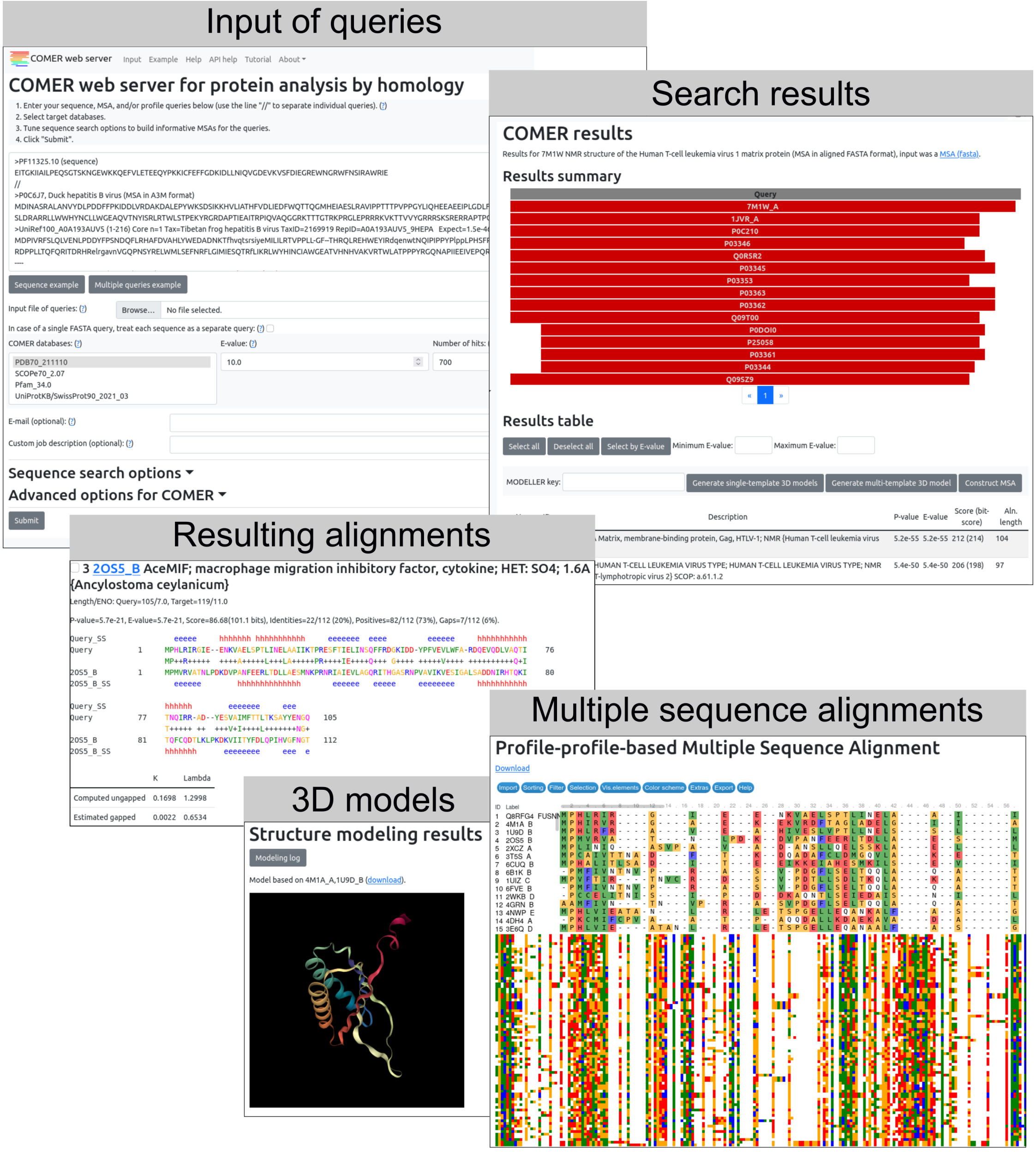


Figure S1. **Web server environment.** Top left: Input form. Top right: Summary section of graphically displayed results. Middle: Resulting pairwise profile-profile alignments. Bottom left: Visual layer to interactively analyze generated structural models. Bottom right: Graphical representation of an MSA built based on resulting profile-profile alignments.

***S2.2.2 Services at the sequence, structure, and function levels*** The web server provides services for protein analysis at various levels. At the sequence level, alignments that COMER2 produces can be selected individually or as a group to build an MSA based on accurate profile-profile alignments. The latter option is particularly useful when the number of produced alignments is large, and only their subset with an $E$-value within a given interval is of interest. Visualizing a resulting MSA using MSAViewer (Yachdav *et al.*, 2016) allows for the interactive analysis of aligned sequences.

Structural analysis is possible through 3D structural model generation with MODELLER (Webb and Sali, 2016) using the structures of identified proteins as templates and produced alignments as restraints. A multiple selection option allows the user to generate one model using multiple templates (up to seven) or multiple models (currently up to 15), one for each selected alignment, with one click of a button. Interactive visual analysis of generated models is maintained using the NGL viewer (Rose *et al.*, 2018) (Figure S1).

The functional inference is based on the results of searching annotated databases. For example, a statistically significant alignment between a query and a protein from the deeply annotated UniProtKB/Swiss-Prot database supplemented with SS predictions may provide strong evidence of a functional relationship. We present an example study of protein annotation below. Generally, simultaneous searching across all available profile databases enables simultaneous analysis of the proteins of interest at the sequence, structure, and function levels.

***S2.2.3 Switching between jobs*** The server keeps track of the user's recent jobs so that the user can submit multiple jobs and conveniently navigate among them.

***S2.2.4 Data availability*** The web server automates homology searches and the MSA and 3D model building processes. The final results of these jobs are available for download once a job is finished. If it is a homology search job, final profile-profile alignments and intermediate results, including initial queries, constructed MSAs and profiles, and log files, will also be available as a single compressed file. These data can be useful for offline analysis and application.

### S2.3 RESTful API

A beneficial feature of the COMER web server is command-line and programmatic access to running homology searches using its computational resources. The communication between the client (the user side) and the server is implemented asynchronously. The advantage is that connections are non-blocking and facilitate the development of automatic workflows on the user side using a simple protocol for communication with the server. A command-line and a Python script example of asynchronously conducting a search can be found on the API help page of the COMER web server. The client communicates with the server using a job identifier, which is returned when initiating a job. These job identifiers are also valid for interactively inspecting jobs in the graphical environment.

## S3 Benchmarking the COMER web server

### S3.1 Comparison with HHpred and HMMER web services

To compare the COMER web server (COMER-WS) with the HHpred service provided by the MPI Bioinformatics Toolkit (Gabler *et al.*, 2020) and the HMMER web server (Potter *et al.*, 2018), we submitted the same set of 100 sequence queries to each server and evaluated their sensitivity, precision, alignment quality, and total execution time.

The database searched was the same for each service: SCOPe 2.07 filtered to 70% sequence identity (SCOPe70_2_07). The HMMER web server does not include a SCOPe database. Therefore, the HMMER3 (v3.3) search for qualitative analysis was performed locally (details to follow).

The diverse set of queries shared no more than 20% sequence identity. Each query represented a distinct SCOPe 2.03 fold. The queries were evenly distributed across the a, b, c, d, e, and f SCOPe classes. The corresponding query sequences were randomly selected from the COMER2 test set (Margelevičius, 2020). Queries, scripts, and output data are available (Software and data availability).

The queries were submitted using the default server settings, except that the maximum number of output alignments for HHpred ("Max targets hits") was increased to 1000. COMER-WS was tested using 2 (default) and 3 HHblits iterations for MSA generation. The resulting MSAs obtained after 3 iterations of HHblits were also used to construct profile hidden Markov models (HMMs) and search the SCOPe70_2_07 sequence database using the hmmsearch program from the HMMER3 software package.

For sensitivity (recall) and precision analysis, a true positive (TP) was a pair of aligned domains that belonged to the same SCOPe superfamily or shared statistically significant structural similarity, i.e., DALI Z-score $> 2$ (Holm *et al.*, 2008). (Alignments between the same domains were removed.) Structurally dissimilar pairs from the same fold were considered to have an unknown relationship and were ignored. Other aligned pairs were false positives (FPs). Recall was calculated as $\mathrm{TP}(s)/\mathrm{P}$; precision was $\mathrm{TP}(s)/(\mathrm{TP}(s) + \mathrm{FP}(s))$, where P is the total number of TPs, and $\mathrm{TP}(s)$ and $\mathrm{FP}(s)$ are the numbers of TPs and FPs evaluated for a threshold value $s$ of a method's statistical significance measure. (The HHpred alignments were sorted by Probability.)

Alignment quality was evaluated by generating structural models based on produced alignments using MODELLER (Webb and Sali, 2016). An alignment was considered to be of high quality (HQA) if the most accurate structural model generated for one of two domains, using the other domain as a template, was significantly similar to the real structure, i.e., TM-score $\geq 0.4$ (and TM-score $\geq 0.5$) (Zhang and Skolnick, 2004). A TM-score $< 0.2$ implied a low-quality alignment (LQA). Alignments were evaluated along the extent of the alignment (local evaluation mode) and with respect to the whole protein domain (global evaluation mode). We also performed precision and recall analysis, assuming HQAs as TPs and LQAs as FPs.

The results, shown in Figure S2, indicate that COMER-WS is more sensitive and produces more HQAs than the other tested methods. The results of large-scale benchmark tests using the same set of input MSAs for each tested method are provided elsewhere (Margelevičius, 2020).

The total execution time of each service is shown in Table S1. COMER-WS permits submitting multiple queries simultaneously. Therefore, Table S1 provides durations for single (#submissions=1) and multiple submissions (#submissions>1) with the total number of queries equal to 100.

HHpred and HMMER do not permit the simultaneous submission of multiple queries, and the queries were submitted one by one (#submissions=100). (HMMER and COMER-WS provide API for command-line and programmatic access, but the purpose of this section is to evaluate interactive performance.) Even though HHpred uses a computer cluster to run many processes

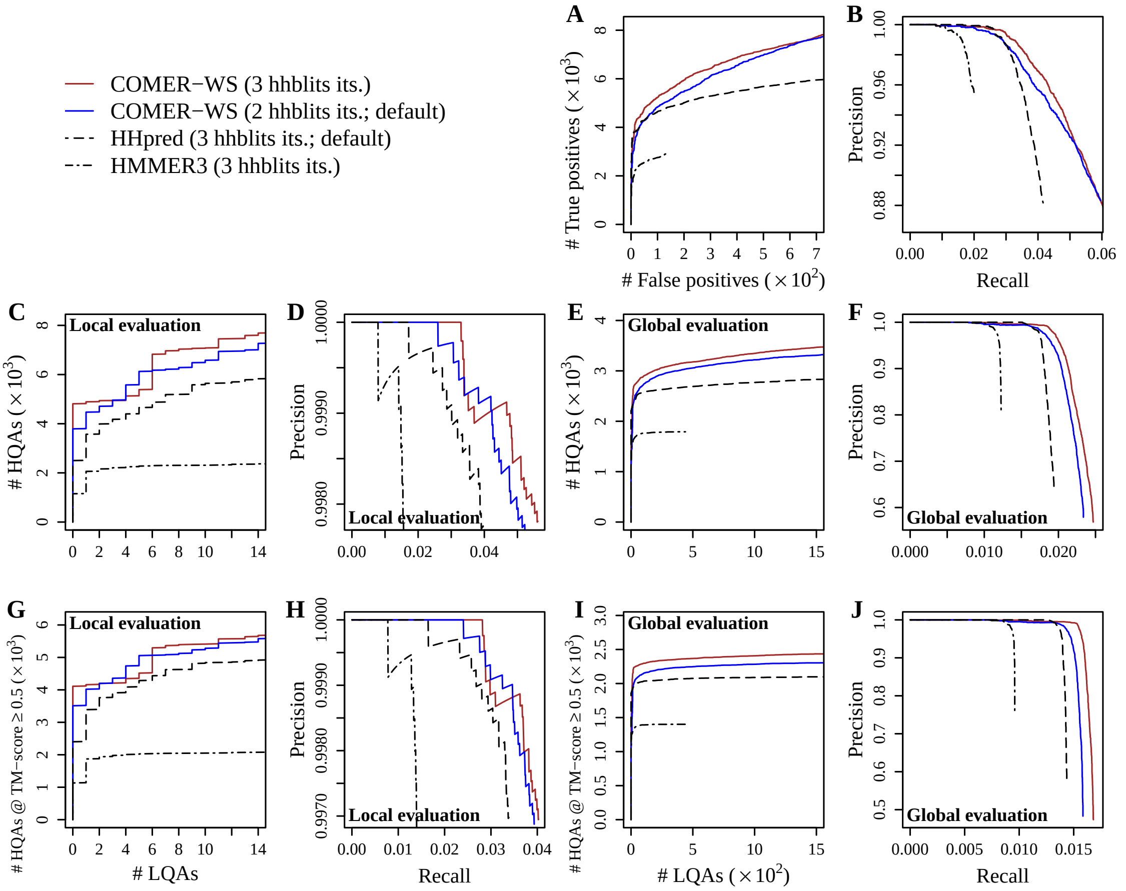


Figure S2. **Benchmarking results: Sensitivity, precision, and alignment quality.** (A) Sensitivity. (B) Precision-recall plot. (C) Local evaluation of alignment quality (HQA: TM-score $\geq$ 0.4). (D) Precision-recall plot for locally evaluated alignment quality (TP $\equiv$ HQA; HQA: TM-score $\geq$ 0.4). (E) Global evaluation of alignment quality (HQA: TM-score $\geq$ 0.4). (F) Precision-recall plot for globally evaluated alignment quality (TP $\equiv$ HQA; HQA: TM-score $\geq$ 0.4). (G) Local evaluation of alignment quality (HQA: TM-score $\geq$ 0.5). (H) Precision-recall plot for locally evaluated alignment quality (TP $\equiv$ HQA; HQA: TM-score $\geq$ 0.5). (I) Global evaluation of alignment quality (HQA: TM-score $\geq$ 0.5). (J) Precision-recall plot for globally evaluated alignment quality (TP $\equiv$ HQA; HQA: TM-score $\geq$ 0.5).

(jobs) in parallel, and a HMMER search takes several seconds (excluding the time taken to generate MSAs), this one-by-one submission by uploading query files prepared in advance (the fastest submission) led to prolonged execution. Downloading the results was also inconvenient (it took another dozen minutes).

The submissions to COMER-WS took almost no effort. However, the current hardware setup does not support full parallelization of all 100 queries. In this case, submitting the queries in groups of 20 was more efficient because the five submissions were processed in parallel. A COMER2 search with all 100 queries took 27 seconds.

| Web server | # submissions | # queries per submission | Total execution time (min) |
|---|---|---|---|
| COMER-WS | 1 | 100 | 144 |
| COMER-WS | 2 | 50 | 78 |
| COMER-WS | 5 | 20 | 43 |
| COMER-WS (3 hhblits its.) | 1 | 100 | 201 |
| COMER-WS (3 hhblits its.) | 2 | 50 | 111 |
| COMER-WS (3 hhblits its.) | 5 | 20 | 61 |
| HHpred | 100 | 1 | 32 |
| HMMER | 100 | 1 | 30 |

Table S1. **Benchmarking results: Total execution time.** Shown are the number of submissions (#submissions), the number of queries per submission (#queries per submission), and the total execution time in minutes for each tested service. COMER-WS was tested using 2 (default) and 3 HHblits iterations for MSA generation. Total execution time includes the time required to submit the queries. HHpred and HMMER do not support multiple simultaneous submissions (hence, #submissions=100).

### S3.2 Execution duration

This section provides the execution times of the COMER web server (COMER-WS) for 21 sequences randomly selected from the UniProtKB/Swiss-Prot90 database. The lengths of the sequences distributed around these values: 100, 200, 500, 1000, 2000, 5000, and 9000. Since COMER-WS limits the maximum query length to 9999, one of the sequences (Q8I3Z1 of length 10,061) was truncated to this length.

Tables S2 and S3 show the execution times for various settings when conducting COMER2 searches against the UniProtKB/Swiss-Prot90 and PDB70 profile databases, respectively. These times were obtained using the server's API. Another dozen seconds would be necessary to render results in the graphical user interface.

The data show that the fastest way for the user to obtain results is to submit profile or MSA queries and skip HHblits and HMMER searches in sequence databases. For example, profile queries are useful for COMER2 searches against updated databases with profiles saved from a previous session. However, the more common case is sequence queries, for which searches against UniRef30 using HHblits (default) for building an MSA take the least time.

Very long sequence queries configured for HMMER searches in the MSA building phase take the longest time. In this case, the user is recommended to modestly use multiple queries for their job (submission) because a job's maximum duration is limited to 24 hours (across all queries of the job). When this time limit is reached, a job will be canceled, and the user will be notified of this event.

| **UniProtKB/Swiss-Prot90** searches | | | | | | | | |
|---|---|---|---|---|---|---|---|---|
| | | Execution time (sec) | | | | | | |
| Seq. ID | Length | Profile query | MSA query | HHblits+ UniRef30 | HHblits+ BFD | HMMER+ UniRef50 | HMMER+ MGnify |
| Q27YE2 | 101 | 17 | 19 | 73 | 121 | 217 | 1351 |
| Q05239 | 101 | 18 | 20 | 74 | 125 | 217 | 1344 |
| P29069 | 101 | 17 | 19 | 69 | 115 | 217 | 1356 |
| A4PBQ0 | 206 | 16 | 20 | 82 | 138 | 233 | 1356 |
| P19743 | 206 | 16 | 20 | 80 | 134 | 216 | 1358 |
| Q86W67 | 206 | 16 | 20 | 80 | 136 | 217 | 1346 |
| O94577 | 502 | 22 | 30 | 120 | 189 | 333 | 1600 |
| Q6GZV8 | 502 | 24 | 30 | 118 | 196 | 228 | 1713 |
| P52638 | 503 | 23 | 29 | 100 | 198 | 228 | 1338 |
| P50535 | 1039 | 40 | 52 | 272 | 439 | 1221 | 2844 |
| Q0WVX5 | 1040 | 45 | 216* | 920 | 1612 | 1475 | 3967 |
| P42835 | 1041 | 40 | 52 | 226 | 372 | 623 | 2168 |
| Q6UDF2 | 2033 | 75 | 99 | 399 | 586 | 755 | 3865 |
| Q54GV0 | 2036 | 73 | 97 | 525 | 864 | 5550 | 12240 |
| Q92576 | 2039 | 78 | 219 | 797 | 766 | 3013 | 6190 |
| A2AAE1 | 5005 | 163 | 447 | 1285 | 1456 | 5133 | 10240 |
| Q9SRU2 | 5098 | 198 | 534 | 1617 | 1617 | 5373 | 13979 |
| Q8SX83 | 5560 | 231 | 291 | 1086 | 1791 | 32106 | 39097 |
| Q9N4M4 | 8545 | 394 | 1555* | 5644 | 5252 | 21185 | 18931 |
| W6RTA4 | 8922 | 352 | 1182* | 4847 | 3407 | 28727 | 17752 |
| Q8I3Z1# | 9999 | 345 | 683 | 2452 | 4956 | 61335 | 58382 |

Table S2. **COMER-WS execution time for UniProtKB/Swiss-Prot90 profile database searches.** The queries correspond to UniProtKB/Swiss-Prot90 sequences (Seq. ID). Six different searches were conducted for each query. The sequences were searched against the HHsuite databases UniRef30 and BFD using HHblits and against the sequence databases UniRef50 and MGnify using HMMER. MSAs obtained from the HHblits search against UniRef30 (MSA query) and COMER2 profiles constructed from these MSAs (Profile query) were used separately to query the server instructed to skip sequence searches. All other settings were set to default values. *MSA reduced to a maximum size of 50MB; #truncated sequence.

| **PDB70** searches | | | | | | | |
|---|---|---|---|---|---|---|---|
| | | Execution time (sec) | | | | | |
| Seq. ID | Length | Profile query | MSA query | HHblits+ UniRef30 | HHblits+ BFD | HMMER+ UniRef50 | HMMER+ MGnify |
| Q27YE2 | 101 | 9 | 12 | 66 | 113 | 209 | 1274 |
| Q05239 | 101 | 9 | 12 | 66 | 118 | 210 | 1268 |
| P29069 | 101 | 9 | 11 | 61 | 108 | 210 | 1280 |
| A4PBQ0 | 206 | 9 | 13 | 75 | 130 | 225 | 1276 |
| P19743 | 206 | 8 | 13 | 73 | 126 | 208 | 1279 |
| Q86W67 | 206 | 8 | 12 | 72 | 128 | 208 | 1266 |
| O94577 | 502 | 8 | 14 | 104 | 174 | 318 | 1513 |
| Q6GZV8 | 502 | 8 | 14 | 102 | 181 | 213 | 1626 |
| P52638 | 503 | 8 | 15 | 86 | 183 | 212 | 1250 |
| P50535 | 1039 | 10 | 22 | 242 | 409 | 1189 | 2746 |
| Q0WVX5 | 1040 | 11 | 178* | 882 | 1578 | 1440 | 3870 |
| P42835 | 1041 | 10 | 22 | 196 | 342 | 592 | 2070 |
| Q6UDF2 | 2033 | 14 | 38 | 338 | 525 | 694 | 3773 |
| Q54GV0 | 2036 | 14 | 38 | 466 | 805 | 5493 | 12149 |
| Q92576 | 2039 | 15 | 159 | 737 | 703 | 2948 | 6099 |
| A2AAE1 | 5005 | 26 | 326 | 1164 | 1317 | 4992 | 10103 |
| Q9SRU2 | 5098 | 33 | 382 | 1465 | 1455 | 5202 | 13806 |
| Q8SX83 | 5560 | 33 | 93 | 888 | 1582 | 31881 | 38869 |
| Q9N4M4 | 8545 | 58 | 1206* | 5295 | 4914 | 20828 | 18636 |
| W6RTA4 | 8922 | 51 | 876* | 4541 | 3104 | 28415 | 17445 |
| Q8I3Z1# | 9999 | 49 | 388 | 2157 | 4656 | 61031 | 58086 |

Table S3. **COMER-WS execution time for PDB70 profile database searches.** See Table S2 for a description. *MSA reduced to a maximum size of 50MB; #truncated sequence.

## S4 Rapid extensive annotation of proteins of unknown function

In this section, we demonstrate protein annotation supported by the COMER web server. For this purpose, all 4730 families of Pfam 34.0 domains of unknown function (DUFs) were searched in the UniProtKB/Swiss-Prot90 (2021_03) profile database using COMER2 with default parameter values. The search on two NVIDIA Tesla V100 GPUs took 211 minutes.

Since HMMER3 is the basic tool for constructing the Pfam database (Mistry *et al.*, 2021), we also searched the corresponding UniProtKB/Swiss-Prot90 sequence database with the profile HMMs of the DUF families using the hmmsearch program from the HMMER3 (Eddy, 2011) software package. The hmmsearch search on all 16 physical cores of two Intel Xeon Gold 5217 CPUs, clocked at 3GHz, took 65 minutes.

We analyzed the distribution of statistically significantly ($E$-value $\leq 0.01$) identified database representatives (significant hits) and the number of unique GO (Gene Ontology Consortium, 2021) Cellular Component, Molecular Function, and Biological Process terms associated with each DUF family through identified significant hits. The results are shown in Figure S3.

COMER2 produced 74,506 significant alignments in total, as opposed to 13,579 significant alignments produced by HMMER3. Both methods produced at least one significant alignment for 2155 DUF families (45.6%), and no significant hits were detected by either method for 1856 DUF families (39.2%). 505 DUF families (10.7%) had significant hits using COMER2 but not HMMER3. COMER2 did not produce significant alignments for 214 DUF families (4.5%), for which HMMER3 did. The analysis of most significant such HMMER3 hits revealed that the corresponding DUF families were short, composed of few sequences, or both, and some of them (e.g., PF06740, a short repeat) were aligned with long database sequences.

COMER2 yielded more than one significant hit for a major part of the DUF families. The number of significant hits correlates with the number of unique GO Cellular Component, Molecular Function, and Biological Process terms calculated for each DUF family across all significant hits, as shown in Figure S3 (the distribution of GO Cellular Component terms is very similar and thus not shown).

We evaluated how closely the hits that COMER2 significantly identified were related to a query DUF family with the maximum number of hits. Specifically, by searching the SCOPe 2.07 profile database, we recorded the most significant match of a DUF family to a SCOPe family of protein domains and calculated how many significant Swiss-Prot90 hits identified for that DUF family also had significant matches to the same SCOPe family. The result for DUF family PF05673 is that all 700 significant hits and PF05673 itself had top significant matches to the same SCOPe family (c.37.1.20). The alignment between PF05673 and its top Swiss-Prot90 hit had medium significance ($E$-value $= 6 \times 10^{-20}$). Considering DUF family PF18949, 566 out of 700 significant hits and PF18949 itself had significant matches to the same SCOPe family (f.24.1.1). The alignment between PF18949 and its top Swiss-Prot90 hit had low significance ($E$-value $= 2 \times 10^{-6}$). Similar results were observed for other analyzed DUF families.

We repeated the same procedure for the HMMER3 insignificant hits ($E$-value $> 0.01$) of several DUF families to make sure that the chosen significance threshold was not too low. For DUF family PF09863, the top matches of 14 HMMER3 insignificant hits distributed across 8 SCOPe folds and 3 classes when searched using HMMER3. PF09863 itself had no match to SCOPe. And only 2 of these HMMER3 hits had top matches to the same SCOPe family (c.1.10.1) as PF09863 did when searched using COMER2. The other two DUF families, PF14054 and PF19795, had no COMER2 or HMMER3 significant matches to SCOPe. For these families, the top matches of 9 and 59 HMMER3 insignificant hits distributed across 7 and 19 SCOPe folds and 4 and 5 classes, respectively. The results of this analysis confirm the unreliability of insignificant

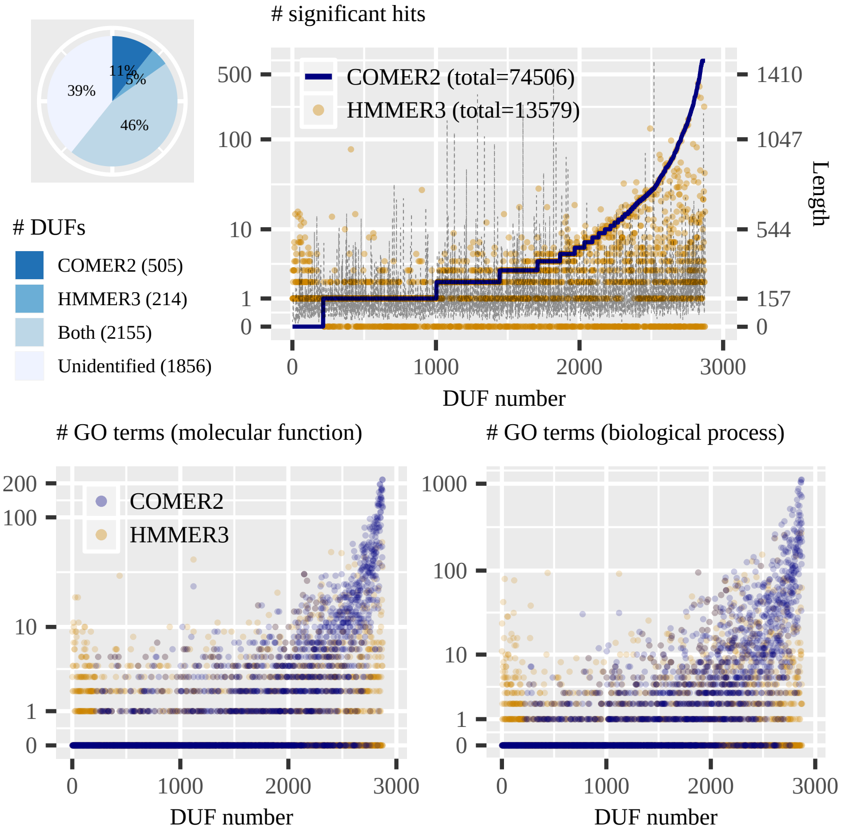


Figure S3. **Annotation of the Pfam 34.0 DUF families.** Top left: Statistics for the number of DUF families. COMER2 (HMMER3): Number of DUF families for which at least one hit was identified using COMER2 (HMMER3) but not HMMER3 (COMER2). Both: Number of DUF families for which both methods produced significant hits. Unidentified: Number of DUF families with no significant hits detected by either method. Top right: Distribution of the number of significant hits identified by COMER2 and HMMER3 (left axis, log scale) for each DUF family. The DUF families are sorted by the number of significant hits identified by COMER2. The dashed line shows the distribution of the DUF family lengths (right axis). Bottom: Distribution of the number of unique GO Molecular Function (left, log scale) and Biological Process (right, log scale) terms calculated for each DUF family across all significant hits. The DUF families are sorted by the number of significant hits identified by COMER2.

hits.

This annotation study demonstrated that COMER2 produces many significant alignments and a relatively large number of them represent true relationships. Consequently, COMER2's sensitive profile-profile comparison is complementary to and may be useful in protein functional annotation.

The COMER2 search results for the 4730 Pfam 34.0 DUF families and accompanying data are publicly available (Software and data availability).

## S5 Case studies

Here, we provide two non-trivial homology examples from the annotation study of proteins of unknown function (Section S4).

### S5.1 PF11821

The most significant hit identified by COMER2 with an $E$-value $= 1.6 \times 10^{-7}$ for DUF family PF11821 (Pfam 34.0) when searching the UniProtKB/Swiss-Prot90 (2021_03) database was P23461 (6% sequence identity)—Rhodobacter capsulatus protein PucD. The search took 37 seconds to run on the COMER web server.

The HMMER3 search (hmmsearch) did not produce significant alignments for PF11821. A hit to P23461 was also missing in the HHblits results obtained from 1 and 3 iterations of the UniRef30 (2022_02) database search with the PF11821 family. (The P23461 entry was in the UniRef30 database.)

In Pfam 35.0, PF11821 was reclassified as the ActD subunit (PDB ID: 6btm_D) of the Alternative complex III (ACIII). Indeed, 6btm_D was the most significant hit ($E$-value $= 1.9 \times 10^{-29}$) obtained by searching the PDB70 (22/07/17) database using COMER2 (or COMER-WS).

ACIII is a key component of bacteria respiratory and/or photosynthetic electron transport chains (Sun *et al.*, 2018). The function of the ActD subunit is unknown, but its structure (a globular domain with two transmembrane alpha helices; Figure S4) and its interaction with other subunits suggest that it may help stabilize the complex.

PucD is a subunit of the LHII light-harvesting complex of the photosynthetic membranes of purple bacteria (Savage *et al.*, 1996), involved in the electron transport chain (Koepke *et al.*, 1996). The function of PucD is unknown, but experiments have shown it to be involved in stabilizing the LHII complex (Weber *et al.*, 1999).

The AlphaFold2 model (Varadi *et al.*, 2022) of PucD shows the same fold as ActD, where ActD has an insertion of two transmembrane helices (Figure S4). The structural and molecular context similarity suggests that the relationship between ActD (PF11821) and PucD (P23461) identified by COMER2 is homologous.

### S5.2 PF09196

In Pfam 34.0 and 35.0, the PF09196 family consists of a single sequence, a Sulfolobus tokodaii maltooligosyl trehalose synthase domain (PDB ID: 3hje:642–704) with an unknown function (Figure S5). COMER2 and HMMER3 for it did not produce significant hits to the UniProtKB/Swiss-Prot90 (2021_03) database.

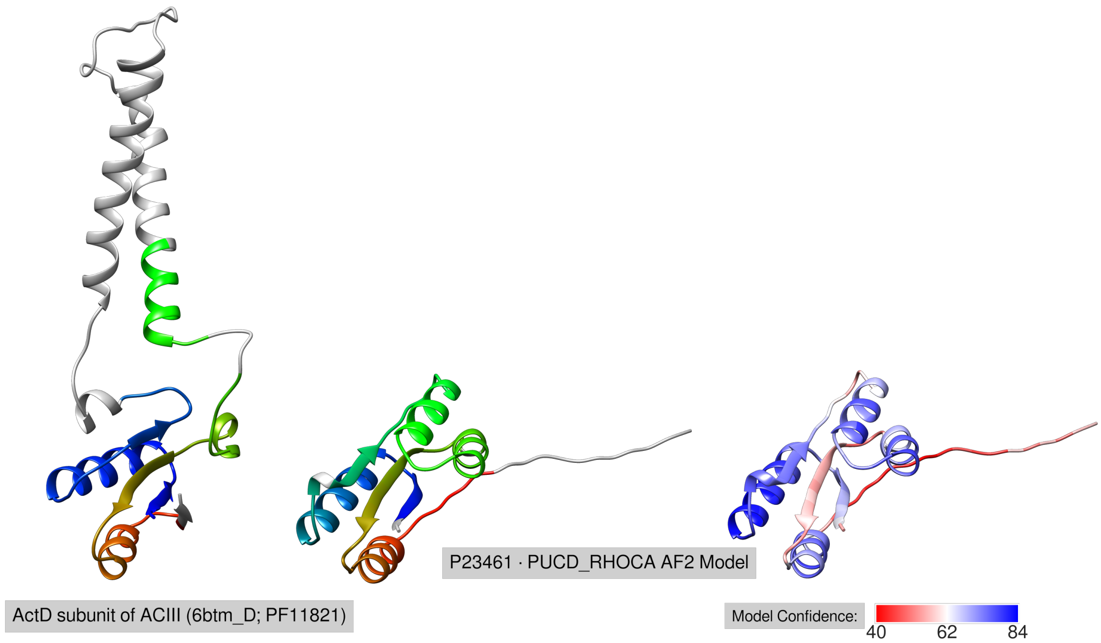


Figure S4. **Structural similarity between ActD (PF11821; left) and the AlphaFold2 model of PucD (P23461; center and right).** The segments aligned by COMER2 are colored blue to red from N- to C-terminus (left and center). The confidence of the AlphaFold2 model is shown on the right. Images prepared using UCSF Chimera (Pettersen *et al.*, 2004).

We conducted 3 HMMER3 iterations against the MGnify sequence database to obtain a more informative MSA (29 sequences) for PF09196, constructed a COMER2 profile, and repeated a COMER2 search against the Swiss-Prot90 database. The whole procedure took 36 minutes to run on the COMER web server.

This time, the most significant was the alignment ($E$-value = 0.0011; 24% sequence identity) with an annotated entry P9WQ20, Mycobacterium tuberculosis putative maltooligosyl trehalose synthase. COMER2 aligned the corresponding domain of P9WQ20 that shares the same fold with PF09196 (Figure S5). The high overall structural similarity of 3hje and P9WQ20 (TM-score = 0.947) also suggests that the two share a common functional mechanism.

The results of 1 and 3 HHblits iterations of the UniRef30 (2022_02) database search did not include hits to P9WQ20 and all its sequence neighbors.

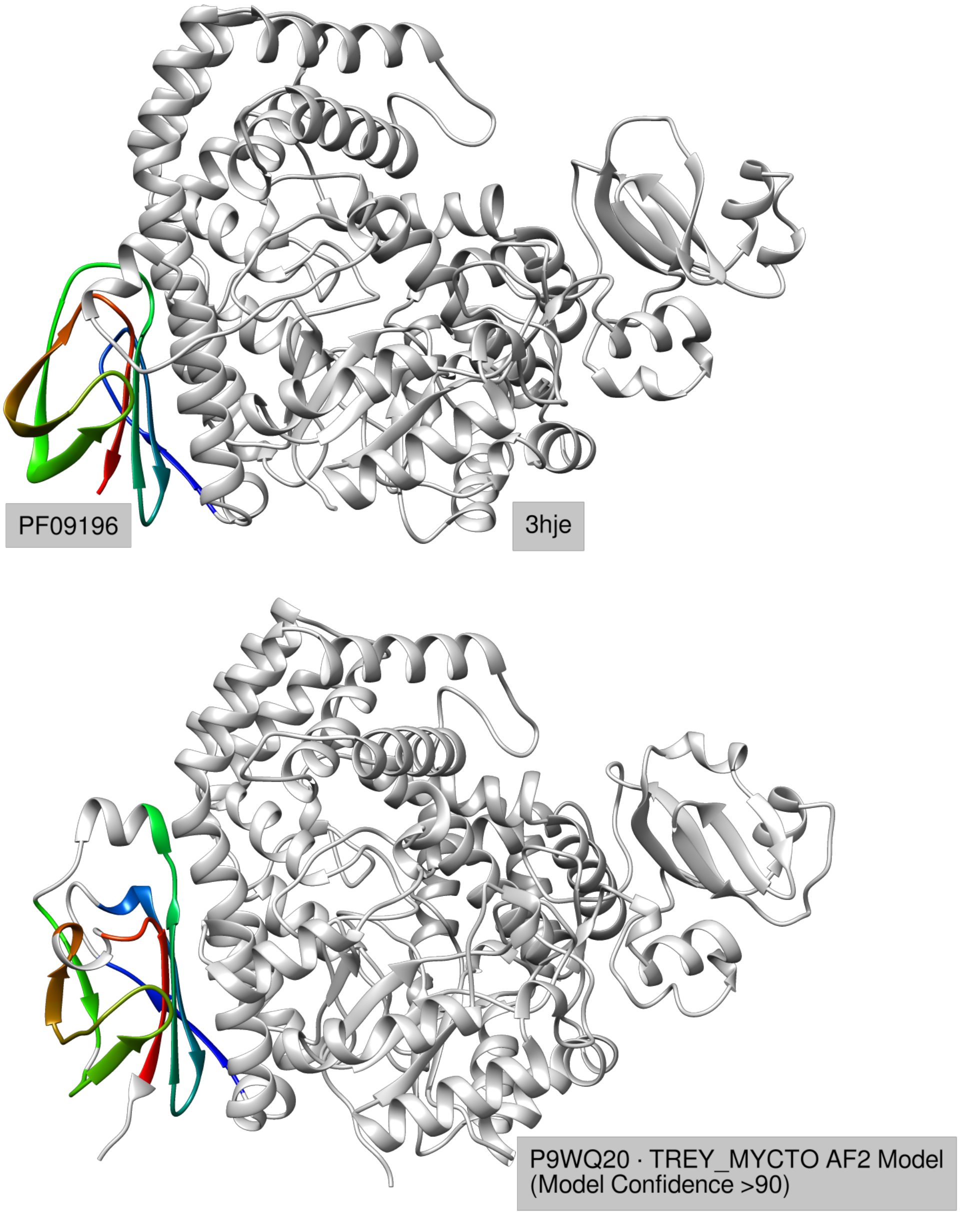


Figure S5. **Structural similarity between PF09196 (colored; top) and the corresponding domain of the AlphaFold2 model of P9WQ20 (colored; bottom).** The segments aligned by COMER2 are colored blue to red. Images prepared using UCSF Chimera (Pettersen *et al.*, 2004).

## S6 Software and data availability

The COMER web server is fully open source. The source code of the web server backend and frontend is available at https://github.com/minmarg/comer-ws-backend and https://github.com/chemikeris/comer_web

Our component tools are open source too and available for download as stand-alone software for local use. The COMER2 software is available at https://github.com/minmarg/comer2 Links to the latest COMER2 profile databases are provided on its website.

The benchmarking data and scripts are available at https://sourceforge.net/projects/comer2/files/comerws-pub-data The COMER2 results obtained using 2 and 3 HHblits iterations for MSA generation are available as completed jobs: https://bioinformatics.lt/comer/search/results/benchmark2 and https://bioinformatics.lt/comer/search/results/benchmark3

The annotation data for the 4730 families of Pfam 34.0 DUFs are available at https://sourceforge.net/projects/comer2/files/comer2-Pfam34-DUF-annotation

## References


Altschul, S. F., Madden, T. L., Schäffer, A. A., Zhang, J., Zhang, Z., Miller, W., and Lipman, D. J. (1997). Gapped BLAST and PSI-BLAST: a new generation of protein database search programs. *Nucleic Acids Res*, **25**(17), 3389–3402.

Burley, S., Bhikadiya, C., Bi, C., Bittrich, S., Chen, L., Crichlow, G., Christie, C., Dalenberg, K., Di Costanzo, L., Duarte, J., *et al.* (2020). RCSB Protein Data Bank: powerful new tools for exploring 3d structures of biological macromolecules for basic and applied research and education in fundamental biology, biomedicine, biotechnology, bioengineering and energy sciences. *Nucleic Acids Res.*, **49**(D1), D437–D451.

Chandonia, J., Fox, N., and Brenner, S. (2019). SCOPe: classification of large macromolecular structures in the structural classification of proteins—extended database. *Nucleic Acids Res.*, **47**(D1), D475–D481.

Eddy, S. (2011). Accelerated profile HMM searches. *PLoS Comput. Biol.*, **7**(10), e1002195.

Gabler, F., Nam, S., Till, S., Mirdita, M., Steinegger, M., Söding, J., Lupas, A., and Alva, V. (2020). Protein sequence analysis using the MPI Bioinformatics Toolkit. *Curr. Protoc. Bioinformatics*, **72**(1), e108.

Galperin, M., Wolf, Y., Makarova, K., Vera Alvarez, R., Landsman, D., and Koonin, E. (2021). COG database update: focus on microbial diversity, model organisms, and widespread pathogens. *Nucleic Acids Res.*, **49**, D274–D281.

Gene Ontology Consortium (2021). The Gene Ontology resource: enriching a GOld mine. *Nucleic Acids Res.*, **49**(D1), D325–D334.

Holm, L., Kääriäinen, S., Rosenström, P., and Schenkel, A. (2008). Searching protein structure databases with DaliLite v.3. *Bioinformatics*, **24**(23), 2780–2781.

Jones, D. (1999). Protein secondary structure prediction based on position-specific scoring matrices. *J. Mol. Biol.*, **292**(2), 195–202.

Koepke, J., Hu, X., Muenke, C., Schulten, K., and Michel, H. (1996). The crystal structure of the light-harvesting complex II (b800–850) from Rhodospirillum molischianum. *Structure*, **4**(5), 581–597.

Lu, S., Wang, J., Chitsaz, F., Derbyshire, M., Geer, R., Gonzales, N., Gwadz, M., Hurwitz, D., Marchler, G., Song, J., *et al.* (2020). CDD/SPARCLE: the conserved domain database in 2020. *Nucleic Acids Res.*, **48**, D265–D268.

Margelevičius, M. (2019). Estimating statistical significance of local protein profile-profile alignments. *BMC Bioinformatics*, **20**, 419.

Margelevičius, M. (2020). COMER2: GPU-accelerated sensitive and specific homology searches. *Bioinformatics*, **36**(11), 3570–3572.

Mirdita, M., von den Driesch, L., Galiez, C., Martin, M., Söding, J., and Steinegger, M. (2017). Uniclust databases of clustered and deeply annotated protein sequences and alignments. *Nucleic Acids Res.*, **45**(D1), D170–D176.

Mistry, J., Chuguransky, S., Williams, L., Qureshi, M., Salazar, G., Sonnhammer, E., Tosatto, S., Paladin, L., Raj, S., Richardson, L., *et al.* (2021). Pfam: The protein families database in 2021. *Nucleic Acids Res.*, **49**(D1), D412–D419.

Mitchell, A., Almeida, A., Beracochea, M., Boland, M., Burgin, J., Cochrane, G., Crusoe, M., Kale, V., Potter, S., Richardson, L., *et al.* (2020). MGnify: the microbiome analysis resource in 2020. *Nucleic Acids Res.*, **48**(D1), D570–D578.

Pettersen, E., Goddard, T., Huang, C., Couch, G., Greenblatt, D., Meng, E., and Ferrin, T. (2004). UCSF Chimera—a visualization system for exploratory research and analysis. *J. Comput. Chem.*, **25**(13), 1605–1612.

Potter, S., Luciani, A., Eddy, S., Park, Y., Lopez, R., and Finn, R. (2018). HMMER web server: 2018 update. *Nucleic Acids Res.*, **46**(W1), W200–W204.

Remmert, M., Biegert, A., Hauser, A., and Söding, J. (2012). HHblits: lightning-fast iterative protein sequence searching by HMM-HMM alignment. *Nat. Methods*, **9**(2), 173–175.

Rose, A., Bradley, A., Valasatava, Y., Duarte, J., Prlić, A., and Rose, P. (2018). NGL viewer: web-based molecular graphics for large complexes. *Bioinformatics*, **34**(21), 3755–3758.

Savage, H., Cyrklaff, M., Montoya, G., Kühlbrandt, W., and Sinning, I. (1996). Two-dimensional structure of light harvesting complex II (LHII) from the purple bacterium Rhodovulum sulfidophilum and comparison with LHII from Rhodopseudomonas acidophila. *Structure*, **4**(3), 243–252.

Schaeffer, R., Liao, Y., Cheng, H., and Grishin, N. (2017). ECOD: new developments in the evolutionary classification of domains. *Nucleic Acids Res.*, **45**(D1), D296–D302.

Steinegger, M., Mirdita, M., and Söding, J. (2019). Protein-level assembly increases protein sequence recovery from metagenomic samples manyfold. *Nat. Methods*, **16**(7), 603–606.

Sun, C., Benlekbir, S., Venkatakrishnan, P., Wang, Y., Hong, S., Hosler, J., Tajkhorshid, E., Rubinstein, J., and Gennis, R. (2018). Structure of the alternative complex III in a supercomplex with cytochrome oxidase. *Nature*, **557**(7703), 123–126.

Suzek, B., Wang, Y., Huang, H., McGarvey, P., Wu, C., and the UniProt Consortium (2015). UniRef clusters: a comprehensive and scalable alternative for improving sequence similarity searches. *Bioinformatics*, **31**(6), 926–932.

Tatusov, R., Fedorova, N., Jackson, J., Jacobs, A., Kiryutin, B., Koonin, E., Krylov, D., Mazumder, R., Mekhedov, S., Nikolskaya, A., *et al.* (2003). The COG database: an updated version includes eukaryotes. *BMC Bioinformatics*, **4**, 41.

UniProt Consortium (2021). UniProt: the universal protein knowledgebase in 2021. *Nucleic Acids Res.*, **49**(D1), D480–D489.

Varadi, M., Anyango, S., Deshpande, M., Nair, S., Natassia, C., Yordanova, G., Yuan, D., Stroe, O., Wood, G., Laydon, A., *et al.* (2022). AlphaFold Protein Structure Database: massively expanding the structural coverage of protein-sequence space with high-accuracy models. *Nucleic Acids Res.*, **50**, D439–D444.

Webb, B. and Sali, A. (2016). Comparative protein structure modeling using MODELLER. *Curr. Protoc. Bioinformatics*, **54**(1), 5.6.1–5.6.37.

Weber, F., Kortlüke, C., and Drews, G. (1999). The polypeptides pucD and pucE stabilize the LHII (b800–850) light-harvesting complex of rhodobacter capsulatus 37B4 and support an effective assembly. In G. A. Peschek, W. Löffelhardt, and G. Schmetterer, editors, *The Phototrophic Prokaryotes*, pages 159–164, Boston, MA. Springer US.

Yachdav, G., Wilzbach, S. ans Rauscher, B., Sheridan, R., Sillitoe, I., Procter, J., Lewis, S., Rost, B., and Goldberg, T. (2016). MSAViewer: interactive javascript visualization of multiple sequence alignments. *Bioinformatics*, **32**(22), 3501–3503.

Yoo, A., Jette, M., and Grondona, M. (2003). SLURM: Simple Linux Utility for Resource Management. In D. Feitelson, L. Rudolph, and U. Schwiegelshohn, editors, *Job Scheduling Strategies for Parallel Processing*, pages 44–60, Berlin, Heidelberg. Springer Berlin Heidelberg.

Zhang, Y. and Skolnick, J. (2004). Scoring function for automated assessment of protein structure template quality. *Proteins*, **57**(4), 702–710.

Zimmermann, L., Stephens, A., Nam, S., Rau, D., Kübler, J., Lozajic, M., Gabler, F., Söding, J., Lupas, A., and Alva, V. (2018). A completely reimplemented MPI Bioinformatics Toolkit with a new HHpred server at its core. *J. Mol. Biol.*, **430**(15), 2237–2243.
